# The myodural bridge complex: a comprehensive review of morphology, physiology, developmental biology and pathology

**DOI:** 10.3389/fmed.2026.1790220

**Published:** 2026-04-17

**Authors:** Lu Zhang, Xue Song, Cheng Chen, Wei Ma, Jian-Fei Zhang, Nan Zheng, Hong-Jin Sui

**Affiliations:** 1 Department of Anatomy, College of Basic Medicine, Dalian Medical University, Dalian, China; 2 Department of Anatomy, College of Basic Medicine, Dalian University, Dalian, China

**Keywords:** biomechanics, cerebrospinal fluid circulation, comparative anatomy, craniocervical junction disorders, developmental biology, evolutionary adaptation, myodural bridge complex, suboccipital musculature

## Abstract

The myodural bridge complex (MDBC) is a phylogenetically conserved composite anatomical structure that anchors the suboccipital musculature and nuchal ligament to the spinal dura mater (SDM) at the cranio-cervical junction, with species-specific morphological adaptations across vertebrates. Physiological functions of the MDBC include hypothesized mediation of cerebrospinal fluid (CSF) circulation dynamics, stabilizing the SDM during head movements to prevent dural folding, and transmitting proprioceptive signals from suboccipital musculature to the central nervous system. Clinical evidence increasingly links MDBC pathological alterations to a spectrum of cranio-cervical disorders, such as chronic cervicogenic headaches, Chiari malformation Type I, cervicogenic dizziness, and Ehlers-Danlos syndrome-associated symptoms. A comprehensive understanding of MDBC structure and function is critical to unraveling its putative mechanistic role in CSF homeostasis and cranio-cervical biomechanics, thereby providing novel insights for the diagnosis and management of related neurological conditions. This review systematically summarizes current knowledge of the MDBC in terms of its morphology, physiology, developmental biology and pathology.

## 1 Morphological characteristics of the MDB

### 1.1 Identification and validation of the RCPmi-SDM connective tissue bridge

Hack et al. (1) first described a connective tissue bridge between the rectus capitis posterior minor muscle (RCPmi) and the spinal dura mater (SDM) in 1995. Subsequent studies further refined the anatomical details of this structure and expanded its known components. Mitchell et al. (2) reported structural continuity between the nuchal ligament (LN) and the SDM exclusively in parasagittal sections at the C1–C2 levels through dissection in 1998. However, Johnson et al. (3) challenged this finding in 2000 using E12 sheet-plastination and dissection, demonstrating that the LN is primarily distributed along the posterior midline of the middle and lower cervical regions, with only loose connective tissue present in the upper cervical region (atlanto-occipital and atlanto-axial interspaces). These conflicting observations highlight cervical connective tissue complexity, requiring clarification via complementary methods. Dean and Mitchell (4) reconfirmed the LN-SDM connections at these upper cervical intervals via dissection, and additionally characterized a connective tissue bridge between the RCPmi and the posterior atlanto-occipital membrane (PAOM) in 2002. Humphreys et al. (5) further validated both the RCPmi-SDM and LN-SDM connections at the C1–C2 levels by integrating dissection with magnetic resonance imaging (MRI) in 2003. They identified connective tissue bridges linking the RCPmi to the SDM and LN (particularly between the posterior arch of C1 and the spinous process of C2) and observed several fascial extensions from the RCPmi to the LN. These findings collectively confirmed the existence of the myodural bridge (MDB) and indicated that it is a composite structure involving multiple connective tissue components. Hack et al. (6) formally proposed the “myodural bridge (MDB)” concept in 2004. Nash et al. (7) elaborated on the RCPmi fiber orientation and MDB at the atlanto-occipital interspace thereby providing a precise anatomical foundation for subsequent functional analyses in 2005. Demetrious et al. (8) incidentally observed the connective structure between the RCPmi, PAOM, and SDM while assessing traumatic injuries in a case of cervical facet subluxation, which is consistent with the MDB first described by Hack et al. (1) in normal specimens in 1995. This study first confirmed the existence of the MDB via MRI in a traumatic pathological state, indicating that the MDB is an inherent and stable anatomical feature of the human body.

### 1.2 Extension of MDB composition: RCPma and OCI to SDM connections

In 2011, Scali et al. (9) identified fibers on the ventral side of the rectus capitis posterior major muscle (RCPma) that traverse the SDM, and subsequent histological analysis revealed that this connecting myodural bridge (MDB) exhibits tendon-like characteristics (10). Building on this discovery, Pontel et al. (11) further extended the understanding of MDB composition by describing an analogous connective tissue bridge between the ventral side of the obliquus capitis inferior muscle (OCI) and the SDM, with its fibers also extending through the atlanto-axial interspace (12). Together, these findings expanded the known anatomical complexity of the MDB, confirming that it is not limited to the classic RCPmi-SDM connection but rather involves multiple suboccipital muscles.

### 1.3 TBNL-SDM connection, suboccipital secondary terminations and MDBC

Sui et al. (13) employed P45 plastination to investigate the connections between the posterior cervical region and the SDM in 2013. They identified MDBs not only at the atlanto-occipital interspace but also at the atlanto-axial interspace, where the structure was designated as the vertebral dural ligament (VDL) within the vertebral canal. This study broadened the understanding of MDB distribution along the cervical vertebrae, revealing that MDB fibers originate from both suboccipital muscles and the nuchal ligament (LN), linking to the SDM via the VDL and a previously uncharacterized structure termed the “to-be-named ligament (TBNL).” Notably, this marked the first identification of the TBNL, representing a significant advancement in anatomical nomenclature and functional comprehension of the MDB complex. In 2016, Yuan et al. (14) further detailed the anatomical relationship between the MDB and the RCPmi, identifying four distinct termination points of the RCPmi. Yuan et al. (15) reported “second terminations” of the suboccipital muscles and variable types of the TBNL in 2017. These second terminations originated from the RCPmi, RCPma, and OCI muscles, merging at the TBNL with an incidence of 34.29% (varying among different muscles). Additionally, 95% of the arcuate-type TBNLs involved these second terminations attaching at the ligament's turning point. The study proposed that these second terminations contribute to maintaining the arcuate morphology of the TBNL and transmitting tension to the MDB, thereby regulating MDB function. This discovery further elucidated the functional dynamics of the MDB complex. Building on this functional insight, Sun et al. (16) used 3D MRI to evaluate subjects in 2020 and found that individuals with RCPmi-TBNL attachments exhibited significantly smaller RCPmi cross-sectional area (CSA), with larger CSA correlating with male gender. This finding indicates a potential functional relationship between RCPmi CSA and TBNL attachment, warranting further investigation. Subsequently, the fibers of the TBNL were formally designated as the Nuchal Dural Bridge (NDB). Collectively, subsequent studies have characterized the fiber origins of the MDB. Beyond the classic RCPmi-SDM connection, the MDB has been confirmed to involve additional structures including the RCPma, OCI, and LN. Fibers from these components traverse the atlanto-occipital and atlanto-axial interspaces to establish connections with the SDM, further enriching the understanding of the MDB as a multi-component composite structure. In 2018, Zheng et al. (17) identified exclusive RCPmi origins for MDB fibers in the atlanto-occipital interspaces, whereas atlanto-axial MDB fibers mainly stem from the RCPmi, RCPma and OCI muscle. These fibers merge into the VDL to link with the SDM and consist primarily of parallel type I collagen fibers. In 2020, Jiang et al. (18) characterized MDB-SDM fusion patterns in the upper cervical interspaces via scanning electron microscopy. Zheng et al. (19) then put forward the “myodural bridge complex (MDBC)” concept using 3D visualization, confirming the MDB as a multi-component composite structure involving the RCPmi, RCPma, OCI muscles and LN. This concept greatly advances the understanding of the MDB's comprehensive function.

## 2 Comparative anatomy of the MDB

After clarifying the human MDB's mature morphological and structural features, exploring its comparative anatomy in vertebrates is essential for elucidating evolutionary origin and conserved function. Zheng et al. (20) identified RCPmi-SDM connective tissue bridges at the atlanto-occipital interspace in seven mammalian species, proposing the MDB as a homologous organ in mammals. Its conservation across multiple mammalian groups was further verified in equines (21), chiropterans and scandentians (22). Aquatic mammals have evolved two specialized MDB subtypes (occipital dural muscle and classical MDB), which are associated with an extensive epidural venous plexus to regulate diving-related blood circulation (23). Neophocaena phocaenoides has independently developed an occipital-derived MDB muscle, and these modifications are suggested to enable aquatic mammals to optimize cerebrospinal fluid (CSF) circulation by remodeling the MDB to adapt to environmental pressures (24, 25).

Comparative studies have confirmed the presence of the MDB in avian (26–28) and reptilian species (29–31). In avians, MDB fibers originate from the dorsal RCPmi, merging with the dorsal atlanto-occipital membrane (DAOM) through trabecular connections to the SDM; Gallus domesticus exhibits three trabeculae types (faint, mesh, cord-like) between the DAOM and SDM (27). Reptiles possess analogous dense fibrous MDB structures: the Siamese crocodile's MDB extends from the anterior atlas to the anterior C2 edge, with trabecular fibers linking the vertebral canal's posterior wall to the SDM (29). This structure forms an indirect dural connection via the anterior atlas, lacking direct attachment to the SDM itself. Notably, avian and reptilian MDBs exhibit distinct morphological features driven by divergent evolutionary adaptive demands. As endothermic animals, birds rely on flight—a high-energy activity that requires both flexible head-neck movements to adjust flight posture and precise regulation of intracranial homeostasis. The trabecular connections and three trabeculae types in avian MDB enable the uniform transmission of suboccipital muscle tension to the SDM, buffering flight-induced vibrational impacts while accurately modulating CSF flow dynamics. In contrast, most reptiles are ectothermic with low locomotor intensity and limited head-neck mobility. Their indirect MDB connections mediated via the anterior atlas are postulated to fulfill the basic requirement of CSF circulation regulation at minimal energy cost, avoiding excessive dural traction during limited movements.

Among reptiles, snakes display a specialized expanded MDB distribution across the atlanto-occipital, atlanto-axial and multiple intervertebral gaps (V2–V6) (31). In contrast, Grondel et al. (32) challenged this finding, suggesting these intervertebral connections are weak fascial links rather than true MDB structures. Such discrepancies stem from differing research emphases: the former focuses on MDB distribution scope, while the latter prioritizes the structural strength of these connections.

Chen et al. (33) further confirmed MDB-like structures in amphibians and bony fish: dense fibrous tissue links interarcual muscles (IAR) to the SDM in Xenopus laevis, and four fish species show direct somatic muscle anchorage to the vertebral canal membrane. Fish rely on lateral undulatory locomotion, whereas terrestrial vertebrates predominantly perform dorsoventral movements. This locomotor divergence drives MDB structural modification, with the fish MDB-like structure functionally transformed into the mammalian MDB to adapt to terrestrial mechanical demands. This fish structure is termed an “MDB-like structure” due to its functional similarity to the mammalian MDB.

Collectively, the MDB is a phylogenetically conserved and universally present structure in vertebrates, with species-specific morphological adaptations to distinct ecological niches. These adaptive modifications are hypothesizedthese modifications allow aquatic mammals to optimize cerebrospinal fluid (CSF) circulation by remodeling the MDB to serve a conserved core function: the regulation of CSF circulation, highlighting the MDB's essential role in vertebrate evolution.

While these comparative findings across vertebrates are instrumental in understanding the MDB's evolutionary history and conserved function, it is crucial to acknowledge the limitations of translating such data to human pathology. Significant interspecies differences—including variations in posture, craniovertebral morphology, subarachnoid space geometry, and CSF pulsatility—can lead to divergent biomechanical and hydrodynamic outcomes. For example, the biomechanical environment of the MDB in a quadruped is not directly comparable to that in bipedal humans, given the substantial morphological differences in the craniovertebral junction and spinal anatomy between quadrupeds and bipeds (34). Consequently, although animal models provide essential mechanistic insights, these must be interpreted with caution. The specific role of the MDB in human CSF circulation, and its potential involvement in disease, ultimately requires direct confirmation through advanced clinical imaging and targeted interventional studies.

## 3 Physiological functions of the MDBC

### 3.1 One of the power sources for CSF circulation

CSF circulation is often referred to as the third circulation in vertebrates. The classical theory of CSF circulation proposes that CSF produced by choroid plexus in the lateral ventricles passes through the foramina of Monro into the 3rd ventricle, then through the cerebral aqueduct into the fourth ventricle (35). CSF from the fourth ventricle may exit laterally through the foramen of Luschka or medially through the foramen of Magendie into the subarachnoid space (36). CSF eventually enters the blood-stream in the dural venous sinuses via arachnoid granulation.

**Figure 1:**
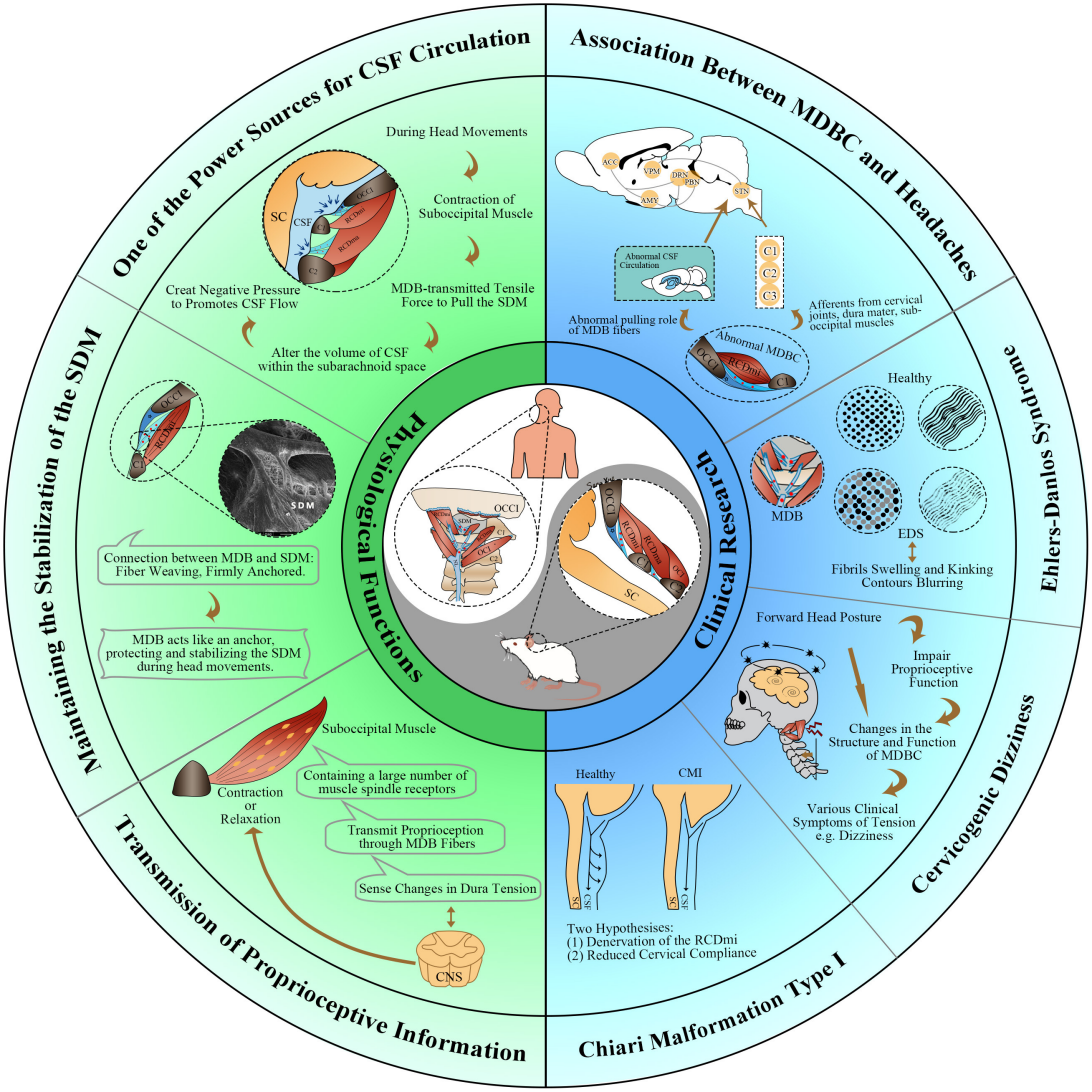
Overview of physiological functions and clinical research of the myodural bridge complex (MDBC). MDB, myodural bridge; MDBC, myodural bridge complex; RCDmi, rectus capitis dorsal minor muscle; RCDma, rectus capitis dorsal major muscle; OCI, obliquus capitis inferior; SC, spinal cord; CSF, cerebrospinal fluid; SDM, spinal dura mater; OCCI, occipital bone; C1, atlas; C2, axis; CNS, central nervous system; EDS, Ehlers-Danlos syndrome; CMI, Chiari malformation Type I.

The dynamic mechanism of CSF circulation has long been a topic of debate within the anatomical community. It is reported that the factors that affect cerebrospinal fluid circulation mainly include (1) Cardiac activity, (2) Respiratory motion, (3) Ciliary movement, (4) Body posture, (5) Intracranial blood circulation (37). In recent years, after more than ten years of dedicated research on MDBC, a research group led by Hong-Jin Sui (13) has proposed and validated a new hypothesis that “MDBC is one of the sources of CSF circulation dynamics.” Subsequently, a series of studies provided experimental evidence to support this hypothesis, shown in Figure 1. The hypothesis points out that the MDBC fibers can transmit tensional forces generated by contraction of suboccipital musculature to the upper cervical SDM during head movements. From a biomechanical perspective, the parallel arrangement of type I collagen fibers endows MDB with effective tensile properties. This transmission may lead to changes in the subdural and subarachnoid volume, potentially creating negative pressure that promotes CSF flow.

With the advancement of research and the continuous development of technology, imaging studies and animal models have been gradually applied to explore the effect of MDBC on CSF circulation (30, 38–43). First of all, preliminary human imaging studies have revealed a correlation between head and neck motion and CSF dynamics. Xu et al. (38) used cine phase-contrast MRI to demonstrate that, after the head rotations, both the maximum and average CSF flow rates during ventricular diastole were significantly increased, as were the CSF stroke volumes during diastole and throughout the entire cardiac cycle; Similarly, after head nodding, both the maximum and average CSF flow rates during ventricular diastole were significantly reduced, and changes in the ratio of cranial and caudal orientation of the net flow volume were significantly different, and the CSF pressure at the L3–L4 level was increased (38). These findings indicate that both flexion-extension and rotational movements of the head can induce changes in cerebrospinal fluid flow at the craniocervical junction.

To further investigate the anatomical basis underlying this association, the researchers conducted a series of animal experiments. Ma et al. (41)investigated the effect of passive head movements in dogs, including lateral flexion, rotation, and flexion-extension. All of these movements resulted in a significant increase in CSF pressure (41). In addition, stimulation of the suboccipital musculature in dogs also led to an increase in CSF pressure; however, this effect disappeared immediately after the severing of the MDB (41). This key finding suggests that the MDB may serve as the structural basis for the mechanical transmission of muscle contraction signals to the SDM. Bruce A. Young et al. (30, 40) found that the American alligator, which possesses a well-developed MDB, showed changes in CSF pressure when the suboccipital musculature were electrically stimulated. The results in the morphological research and electrical stimulation experiment of the suboccipital musculature in American alligator supported two common hypotheses: that the MDB plays a role in altering CSF pressure, or that it adjusts the SDM (30, 40). More specifically, the MDB in alligators functions to regulate dural compliance at the foramen magnum, thereby influencing the dynamics of CSF exchange between the cranial and spinal compartments (44). Studies in a rat model suggest the effects of the MDB on CSF circulation. The model of the suboccipital musculature hypertrophy in rats demonstrated that the hypertrophy of the suboccipital musculature, along with increased muscle contraction strength, could lead to an increase in CSF pressure (42). Subsequent research measured the CSF secretion and reabsorption rates in rat models with suboccipital musculature atrophy or hyperplasia (43). Their findings demonstrated a significant increase in CSF secretion rate in rats with the hyperplasia model, while rats with the atrophy showed a significant decrease in CSF secretion rate (43). Additionally, both the atrophy and hyperplasia groups showed an increase in CSF reabsorption rate (43). To further investigate the potential mechanism by which the MDB modulates cerebrospinal fluid dynamics, Yuan et al. (45) investigated the temporal and spatial variations in CSF pressure during electrical stimulation of the OCI. The suboccipital muscles influence the occipital cistern through the MDB, generating pressure waves that spread through the subarachnoid space and ventricles and maintain local high pressure in the frontal region. Cerebrospinal fluid pressure waves diffuse from the occipito-atlantal cistern to the cranial cavity and spinal canal following stable spatiotemporal patterns, which depend on anatomical structures and the distance from the occipito-atlantal cistern. This mechanism represents one pathway through which the MDBC drives CSF circulation.

Research on the MDB provides a novel perspective for understanding the regulatory mechanisms of CSF circulation. However, as a driving force independent of cardiac and respiratory movements, it still requires validation by more functional studies.

### 3.2 Maintaining the stabilization of the SDM

Anatomical studies have revealed the structural basis for maintaining dural stability at the craniocervical junction. In an earlier study, Hack et al. (1) speculated that this connective tissue bridge of the “PAO membrane-spinal dura complex” may help resist dural infolding during head and neck extension. In a study of 20 cadavers, Nakagawa (46) concluded that function of the various ligaments and the posterior cervical SDM was to resist the forces of hyperextension and compressive folding of the SDM. Recent histological analysis has further indicated that, at the cranio-cervical junction, MDB, occipital periosteum and collagen fibers of the SDM were crossed and fused to form collagen fiberboard layers of the SDM, giving them obvious anisotropic biomechanical properties (47, 48). This characteristic endows it with the capacity to counteract dural tensile and folding stresses induced by diverse head movements in a targeted manner, thereby providing biomechanical plausibility for the functional hypothesis that the MDB maintains SDM stability.

Based on the above anatomical basis, researchers have provided a functional explanation for the mechanical role of the MDB during head movement. During flexion of the head and neck, the atlanto-occipital interspace widens, and the suboccipital musculature tightens the MDB fibers to prevent the folding of the SDM (49). During the extension of the cranio-cervical joint, the RCPmi and RCPma contract to generate tension along the MDB fibers at the atlanto-occipital and atlanto-axial interspaces, which leads to posterior traction of the SDM (49). During the rotation of the atlanto-axial joint, the RCPma and OCI contract and transmit tension through the MDB of the atlanto-axial interspace, which also prevents the folding of the SDM at this level (12, 49). Finite element segmental analysis showed that excessive activity of the upper cervical segments increased the mechanical load on the spinal cord (50). Combined with anatomical observations, it can be speculated that the MDB may exert a protective regulatory effect on the morphological stability of the SDM during head motion by transmitting mechanical activity of the suboccipital muscles to the SDM, shown in Figure 1.

### 3.3 Transmission of proprioceptive information

Muscle spindles are the primary proprioceptive organs, which play a vital role in motor control and musculoskeletal function. There are more muscle spindle receptors in the suboccipital musculature than in other musculature in the body, shown in Figure 1. On average, the suboccipital musculature has 36 muscle spindles per gram, while other musculature in the body, such as the gluteus maximus contains only 0.8 muscle spindles per gram (51, 52). These receptors are essential for conveying proprioceptive information (51). Under normal physiological conditions, proprioceptive information input from cervical muscle spindles and cervical disc and facet joint mechanoreceptors is integrated and transmitted to the central nervous system to control head position, head orientation, and whole-body posture (53–55). When the movement patterns of the atlanto-occipital and atlanto-axial joints change, the suboccipital musculature will quickly adjust to effectively prevent damage to the SDM and spinal cord caused by folding or tension (49).

MDB serves as an important anatomical structure connecting the suboccipital musculature and the SDM. Based on this anatomical relationship, researchers have proposed a functional hypothesis regarding the proprioceptive transmission pathway. The hypothesis states that during movements of the atlanto-occipital and atlanto-axial joints, the tension of the SDM changes. The muscle spindle-rich suboccipital muscles may detect dural tension changes via the MDB fibers and convert these mechanical signals into proprioceptive afferent information. After integration by the central nervous system, such information reflexively regulates the contraction of the suboccipital muscles, thereby maintaining the morphological stability of the SDM and preventing its excessive folding or injury during dynamic motion (49). From a clinical perspective, this hypothesis also suggests potential pathological associations. Any changes in the load or function of the MDB may subsequently impair the transmission of proprioception and muscle control, leading to dural stimulation, alterations in dural tissue compliance, and changes in CSF dynamics. Further validation of this pathological connection through clinical imaging and interventional studies is required.

## 4 Development and regulation of the MDBC

### 4.1 Embryonic and postnatal development

#### 4.1.1 Human

The development of the human MDBC is a dynamic and progressive process from the embryonic period to postnatal stages. José Francisco Rodriguez-Vázquez et al. (56) shows that dense, regular fibrous connective tissue connections integrate into the SDM originated from the PAOM, PAAM, RCPmi, RCPma, OCI, and LN. At 12 weeks of gestation (FW12), the MDBC remains in a primitive state, characterized by loose connective tissue and dispersed myotubes with no obvious structural differentiation (57). At 14–16 weeks of gestation (FW14–16), fetal head movements become more prominent, and the generated mechanical stimuli—including traction from dural-spinal canal separation and tension from suboccipital muscle contractions—act as external stresses to promote the ordered arrangement of MDB fibers and the formation of interconnections (58). This stage initiates functional preparation for MDB-mediated force transmission. At 17–19 weeks of gestation (FW17–19), increased head movement frequency and structural/functional maturation of suboccipital muscles further drive MDB development. By 21 weeks of gestation (FW21), cell differentiation and structural refinement are largely completed. At 25–30 weeks of gestation (FW25–30), NDB fibers originating from the tensed band of the TBNL are first observed. Notably, the MDB originating from the suboccipital muscle is denser and more ordered than the NDB originating from the TBNL level (57). The MDB fibers align along the direction of suboccipital muscle contraction, thus carrying the main mechanical load during muscle contraction. Its high density directly reflects its primary role in regulating dural tension and subsequent CSF circulation dynamics, whereas the NDB with scattered fiber arrangement undertakes a secondary stabilizing role.

At 30–37 weeks of gestation (FW30–37), Kitamura et al. (59) identified two types of candidate MDB structures in fetal specimens: (1) a thick connective tissue band between the RCPma and RCPmi (Type 1, 90%); and (2) a thin fascia extending from the upper margin of the RCPmi (Type 2, 66.7%). Coexistence of multiple origins was found in 90% of the 27 fetal specimens. Postnatally, the MDB matures further with elevated fiber density and improved alignment regularity, culminating in the formation of a robust tendon-like structure that fully adapts to the biomechanical requirements of postnatal head and neck motility (59).

Conflicting views exist regarding the mechanical function of the fetal MDB. Song et al. (57) stated that biomechanical stimuli drive MDB fiber maturation and lay a structural foundation for force transmission. In contrast, Kitamura et al. (59) found that the candidate MDB structures at FW30–37 lack elastic fibers, and the suboccipital muscles exhibit limited force transmission to the SDM at this stage, owing to scattered fiber arrangement, weak muscle-fascia connections and venous plexus interference in the atlanto-occipital region. This contradiction arises primarily from the divergent gestational stages selected by the two research teams, as well as the inherently dynamic developmental pattern of the fetal MDB. Song et al. focused on early-to-mid gestation, while Kitamura et al. investigated late gestation, which represents a transient developmental stage rather than a definitive functional endpoint. During embryonic and post-natal development, most tendons experience an increase in collagen content and a decrease in cellularity (60–62). The immature structural features observed at this late fetal stage do not indicate functional deficiency, as MDB maturation continues postnatally.

Collectively, these findings confirm that the fetal MDB undergoes progressive mechanical adaptive remodeling throughout gestation. This dynamic prenatal-postnatal developmental process ensures the MDB gradually adapts to the increasing biomechanical demands of head and neck movements, highlighting the essential role of mechanical stress in the ontogenetic development of this tendon-like structure.

#### 4.1.2 Rodent

The development of the MDBC was also observed in SD rats (63). At embryonic day 18 (E18), the posterior arch of the atlas is fully closed, MDB fibers initiate formation, and only a small number of fibers and muscular tissues are present in the suboccipital region—a feature that persists until E21, with a marked increase observed postnatally. At P1, MDB fibers originated from the RCDmi and merged into the dorsal atlanto-occipital membrane (DAOM). As rats matured, MDB fibers gradually became denser and more organized. In addition, Lai et al. (64) observed that the connection between the PAAM, MDB, and the SDM within the PAAI was less dense than the connection between the MDB and the SDM within the PAOI in adult rats.

Table 1 summarizes the stage-specific developmental characteristics of the MDBC in humans and rats. Comparative studies on the MDBC development in human and rat embryos have revealed that despite differences in specific developmental timelines and anatomical features, both undergo a transition from primitive, disordered connective tissue to mature, ordered fibrous structures as a highly conserved evolutionary adaptation. This conservation emphasizes the critical biomechanical role of the MDBC in supporting head and neck biomechanical function in vertebrates, while differences in developmental timelines mainly reflect adaptations of different species to specific motor demands and developmental rates.

**Table 1:** Developmental changes in MDBC composition.

	Humans	Rats	Conclusion
Changes in collagen fiber composition of MDB	From FW14: fibers gradually became orderly and parallel. After FW21: primarily composed of type I collagen fibers, arranged in parallel.	Embryonic stage (E18–E21): Contained both type I and type III collagen fibers. Postnatal stage (after P0): Mainly composed of type I collagen fibers, with enhanced red birefringence under polarized light.	As MDB develops, the proportion of type I collagen fibers gradually increases.
Changes in cell type of MDB	FW12–13: Dominated by fibroblasts. After FW21: Gradually differentiated into fibrocytes.	Embryonic stage: Disordered fibroblasts in the PAOM, PAAM. Postnatal stage: Fibrocytes gradually became the dominant cell type.	These transitions reflect MDB maturation, from an immature state (fibroblasts) to a mature state characterized by fibrocytes.
Changes in the suboccipital muscles	FW12: Myotubes and immature muscle fibers with numerous nuclei. FW19: Muscle cells matured, with myofibrils filling the myotubes. After FW21: Muscle structure stabilized.	Embryonic stage (E18–E21): RCDmi muscle fibers were loose and fusiform. Postnatal stage (after P0): Muscle fibers became denser and increased in number.	Muscle fibers are loose in the embryonic stage and gradually become dense and mature after birth.
Changes in the SDM, PAOM, and PAAM	SDM: FW12 thin (fibroblasts) → FW13 thickened (fibrocytes) → gradually denser. PAAM: FW12 completely sealed the atlanto-axial space → FW14 gap appeared at the caudal end. From FW14: Dura separated from spinal canal, with epidural space formed.	SDM: Dense in embryonic stage, separated from spinal cord postnatally. PAOM: Thin in embryonic stage, thickened postnatally and fused with SDM; in Postnatal stage, MDB fibers passed through PAOM to connect with SDM. PAAM: Thin in embryonic stage, thickened postnatally; connection between PAAM, MDB, and SDM was less dense compared to that in the atlanto-occipital interspace.	Dura-related structures (SDM, PAOM, PAAM) all become thicker and denser with development, and their connections with surrounding structures gradually become clear, providing a structural basis for the function of MDB.

MDB, myodural bridge; PAOM, posterior atlanto-occipital membrane; PAAM, posterior atlanto-axial membrane; SDM, spinal dura mater; FW12, fetus at 12 weeks of gestation; FW13, fetus at 13 weeks of gestation; FW14, fetus at 14 weeks of gestation; FW21, fetus at 21 weeks of gestation. E, embryonic (for rat); P, postnatal (for rat); W, week; RCDmi, the rectus capitis dorsal minor muscle.

### 4.2 Potential factors influencing MDBC development

#### 4.2.1 Mechanical properties and adaptation

Type I collagen constitutes the core structural backbone of the MDB and is indispensable for mediating force transmission within the structure. Numerous studies have investigated the regulatory roles of different collagen components and transcriptional regulators in the formation of tendon structure, maintenance of mechanical properties, and repair of injuries. Sun et al. (65) investigated the role of collagen XI in regulating the mechanical properties of tendons by using a tendon-specific Col11a1-null mouse model and analyzing the biomechanical performance of flexor digitorum longus tendons (FDLs) at different developmental stages, and found that tendon-specific deletion of Col11a1 led to abnormal collagen fibril structure (shift to smaller diameters and disrupted parallel alignment), significantly decreased maximum stress and modulus of mature FDLs, and impaired grip strength and gait ability in mice, revealing that collagen XI plays an essential role in regulating tendon fibril assembly and organization during development, which is critical for the acquisition of normal tendon mechanical properties. Collagen V is a critical regulator of collagen I fibrils, Leiphart et al. (66) investigated the role of collagen V in regulating mechanical properties of healing tendons using inducible Col5a1 knockdown models and mechanical analysis of injured patellar tendons, and found that heterozygous Col5a1 knockdown improved healing tendon stiffness and proposed that optimizing quasi-static mechanics of healing tendons may be achieved by modulating collagen V expression to regulate collagen fibril size and shape. Besides, Dingwall et al. (67) investigated the transcriptional and epigenetic regulatory mechanisms underlying the changes in tendon biomechanical properties during postnatal maturation, and found that conditional deletion of Yap1 (a transcriptional co-activator of the Hippo signaling pathway) at postnatal stages altered the early expression of Col1a1 as well as matrix organization and density, but did not affect the gross ultrastructural and overall mechanical properties of tendons at later postnatal stages. Robinson et al. (68) investigated the roles of decorin and biglycan in maintaining the mechanical properties of mature tendons by using an inducible knockout mouse model to ablate the two genes specifically in the mature patellar tendon, and found that acute deletion of decorin and biglycan led to reduced load-bearing capacity, decreased stiffness, increased percent relaxation and tissue viscosity, delayed and slower collagen fiber realignment in response to load, and a trend toward decreased dynamic modulus, demonstrating that these two small leucine-rich proteoglycans play a significant role in the homeostasis and mechanical stability of mature tendons.

The above molecular regulatory mechanisms underlying tendon structure-mechanical property relationships provide a theoretical framework for understanding the mechanical adaptation of the MDB, which shares structural and functional similarities with tendons.

In the musculoskeletal system, tendons are subjected to three main categories of mechanical loading: tension (unidirectional tensile force), compression (force exerted from one or more directions), and shear (force inducing tissue sliding) (69). Tendons developing under tensile loading typically form a dense, well-aligned extracellular matrix dominated by Type I collagen—a trait shared by the MDB, potentially driven by the tensile loads generated during head movements. Functionally, the MDB mediates force transmission among the RCPmi, occipital bone, and SDM, thereby enabling head nodding, shaking, and rotation. Despite these insights, a critical research gap persists: no studies have quantitatively evaluated the mechanical properties of the MDB under dynamic cyclic loading to date.

During development, tendons exhibit significant changes in collagen content and cellularity, which continue into adulthood as tendons respond to varying mechanical environments. These adaptations are driven by intricate molecular and cellular mechanisms, including the synthesis of specific proteins and the modulation of gene expression in response to mechanical stimuli (69).

Mechanotransduction is a critical process that enables tendon cells, such as tenocytes and tendon stem/progenitor cells, to sense and respond to mechanical stimuli, converting external forces into intracellular biochemical signals that regulate cell behavior, matrix remodeling, and tissue homeostasis (70). This process follows a hierarchical signal transduction cascade, starting from membrane receptors to intracellular signaling and transcriptional regulation.

##### 4.2.1.1 Integrins: the primary mechanical sensors at the cell membrane

Integrins, as transmembrane receptors, play a pivotal role in mechanotransduction by linking the extracellular matrix (ECM) to the cytoskeleton and serving as the primary sensors of mechanical forces (71). Mechanotransduction is initiated at the cell membrane, where integrins form complexes with focal adhesion proteins like vinculin, talin, and paxillin, creating a physical bridge between the ECM and the actin cytoskeleton (72). Upon mechanical stimulation, integrins cluster and recruit focal adhesion complexes, including focal adhesion kinase and Src kinases, which initiate downstream signaling cascades (73). This physical coupling allows mechanical signals to be transmitted directly to the cell interior, where they activate intracellular signaling pathways such as the MAPK/ERK, Rho/ROCK, TGFβ/Smad2/3 and PI3K/Akt pathways, which are crucial for regulating gene expression, cytoskeletal dynamics, and protein synthesis (74). Activation of integrin downstream pathways can also lead to the phosphorylation of YAP/TAZ, which translocate to the nucleus and modulate the expression of genes involved in cell proliferation, differentiation, and matrix remodeling (75). The critical role of integrins in mechanotransduction is also validated in the MDB, a specialized structure sharing mechanical adaptation characteristics with tendons. Zhang et al. (76) investigated the morphological changes of the MDB in response to altered mechanical force environments by suppressing Integrin α7 (ITGA7) via shRNA injection into the dorsal atlanto-occipital interspace of neonatal SD rats, and found that ITGA7 knockdown induced significant morphological alterations of the MDB, including disrupted fiber assembly, disorganized collagen fiber architecture (indicative of immature collagen), along with RCDmi muscle dystrophy. The MDB may undergo distinct morphological adaptations in response to changes in its mechanical force environment.

##### 4.2.1.2 Actin cytoskeleton: the structural scaffold for mechanical signal transmission

The actin cytoskeleton plays a crucial role in mechanosensing by providing structural support and regulating the mechanical properties of cells (77). Actin filaments connect integrins to the nucleus through the LINC (linker of nucleoskeleton and cytoskeleton) complex, enabling the transmission of mechanical signals directly to the nuclear lamina (77). This physical coupling allows mechanical signals from integrin complexes to be propagated into the cell interior, serving as a structural scaffold for downstream signaling activation. For example, increased matrix stiffness transmitted via integrins promotes actin cytoskeleton reorganization, which further activates downstream signaling pathways (75). Conversely, a softer ECM inhibits such cytoskeletal rearrangement, leading to reduced signal transduction (78). These adaptive responses of the actin cytoskeleton are essential for translating external mechanical cues into intracellular biochemical signals, bridging membrane receptors and downstream signaling molecules (69).

##### 4.2.1.3 PIEZO1/TRPV4: mechanosensitive ion channels mediating calcium signaling

Among mechanical stress-responsive ion channels, the PIEZO family (PIEZO1 and PIEZO2) and TRPV4 have emerged as key mediators in musculoskeletal tissues, particularly tendons. PIEZO1 and PIEZO2 are mechanically activated Ca^2+^ ion channels (79, 80). PIEZO1 senses shear stresses induced by collagen-fiber sliding in tenocytes, triggering Ca^2+^ influx to initiate downstream signaling (81). A few studies have focused on the role of TRPV4 in the musculoskeletal system, demonstrating that TRPV4 affects the orientation of collagen production by regulating the tensile force of vinculin (a key component of focal adhesion complexes downstream of integrins) in mesenchymal stem cells (82). Notably, TRPV4 interacts with focal adhesion complexes downstream of integrins, forming a synergistic regulatory network for mechanical signal transduction.

##### 4.2.1.4 MKX/YAP/TAZ: transcription factors regulating gene expression and tissue adaptation

The mechanical signals transmitted via integrins, the actin cytoskeleton, and ion channels (PIEZO1/TRPV4) ultimately converge on transcription factors such as MKX, YAP, and TAZ, which modulate gene expression to drive tissue adaptation. Mohawk (MKX), a master transcription factor of tendon development, is a key mediator in tendon mechanotransduction. When tensile stress is applied to tendonstem/progenitorcells derived from the patellar tendon of wild-type rats, the expression of MKX and its downstream tendon-related genes (e.g., Col1a1, various proteoglycans) increases (83, 84). Li et al. (85) found that MKX bidirectionally regulates the development of the MDBC in SD rats through the TGF-β signaling pathway, further confirming its role in mechanosensitive tissue development. The mechanical stress-PIEZO1-MKX/SCX cascade in tenocytes induces structural modification of tendon tissue; transcriptome and immunohistology analysis of Achilles tendons from tendon-specific Piezo1 GOF mice showed increased expression of MKX and SCX, fascicle-forming matrices (including type I collagen), and noncollagenous matrices forming IFM (including Tnmd, Dcn, Fmod, and Prg4) (81, 86). For instance, increased matrix stiffness promotes YAP/TAZ activation, enhancing matrix protein expression and cell proliferation to stiffen the tissue (75), while a softer ECM inhibits YAP/TAZ signaling, reducing matrix synthesis (78).

##### 4.2.1.5 Functional outcomes and pathological implications

Appropriate mechanical stress plays a crucial role in promoting an anabolic effect on tenocytes by regulating the expression of tendon-specific genes via this complete mechanotransduction cascade. Nevertheless, excessive mechanical stress induces tendon degeneration through de-differentiation or inflammation, and nonmechanical stress exerts a catabolic effect on tendons (87). The balance between collagen synthesis and degradation is crucial for maintaining tendon health, and the extent of collagen turnover remains a topic of ongoing research (69).

#### 4.2.2 Age-related changes in structure and function

Feng et al. (88) retrospectively analyzed T2-weighted imaging sagittal images of the cervical region in 1,085 patients. Their results identified four MRI types of MDBC: Type A (no MDBC hyposignal shadow connected to the SDM in either the PAOiS or PAAiS), Type B (MDBC hyposignal shadow connected to the SDM in the PAOiS only), Type C (MDBC hyposignal shadow connected to the SDM in the PAAiS only), and Type D (MDBC hyposignal shadow connected to the SDM in both the PAOiS and PAAiS). In this study, age and cervical degeneration substantially contributed to MDBC imaging staging. The population distribution of MDBC imaging classification changed noticeably with age. The analysis indicated a decrease and increase in the incidence of type B and C MDBC, respectively, with aging. Future research is needed to investigate the mechanisms of MDBC with aging.

Aging has a significant impact on tendons, affecting both their constituent cells and ECM. As a connective tissue whose primary function is to transmit muscle-generated contractile force to bone, the collective actions of tendon cells are important for maintaining the homeostatic state of the body. However, undesired molecular changes to this ECM-rich tissue may negatively influence its mechanical attributes (89, 90).

Tendon fibroblasts from old mice exhibited low motility, a poorly organized actin cytoskeleton, and a different localization of key focal adhesion proteins as compared with young cells (91). The cellular senescence-inhibited gene (CSIG) is expressed abundantly in young tendon fibroblasts, but its expression declines during cellular senescence. In aging tendon the reduction in proliferative capacity is associated with the down-regulation of CSIG and an increase in p27, a cell cycle inhibitor protein. CSIG modulates replicative senescence. A reduction in CSIG reduces cell growth and accelerates cellular senescence (92). Sugiyama et al. reported that in mouse flexor tendons, the mRNA expression levels of collagens (type I and type III) and tenogenic markers (MKX, SCX and TNMD) declined substantially with aging (93). Beyond the reduction in ECM synthesis, the balance of ECM degradation—mediated by multiple remodeling enzymes including matrix metalloproteinases (MMPs) and their inhibitors (TIMPs)—also plays a critical role in maintaining tendon homeostasis (94, 95). Nevertheless, aging-associated changes in collagen structure are expected to significantly impact tendon biomechanical properties, such as decreased tensile strength, altered viscoelasticity, limited fiber sliding, and increased stiffness that were observed during aging (96). Given that the MDBC shares similar fibrous structural characteristics and mechanical adaptation mechanisms with tendons, a deeper understanding of the function and biological characteristics of MDBC is required to investigate the effects of aging at more specific sub-structural levels.

### 4.3 Animal models for validating MDBC function

Experimental animal models are essential tools for studying function of MDBC. By simulating the pathological environment of human MDBC, experimental animal models can help to gain a deeper understanding of the physiological functions of MDBC. Experimental animal models also provide crucial foundation for the original theoretical research supporting the idea that MDBC is one of the sources of CSF circulation dynamics. Table 2 summarizes the research characteristics of animal models related to MDBC in currently published articles. These models provide an alternative animal experimental plan for simulating pathological changes in the MDBC.

**Table 2:** Research characteristics of MDBC related animal models in previous studies.

Models	Method	Animal	Advantage	Limitation	Reference
Suboccipital musculature hyperplasia	Injection of Myostatin-specific inhibitor (ACE-031) into the suboccipital musculature	8w/12w SD male rats	Alteration of MDB-transmitted tensile force induced by pathological changes of the suboccipital musculature may play a potential role in CSF circulation. This model provides strong evidence for the involvement of MDBC in CSF circulation.	(1) No direct method has been found to measure the contractility of RCDma and RCDmi; (2) There is a lack of direct observation of MDB fibers; (3) Due to technical limitations, there is no complete non-invasive measurement of CSF production/absorption rate.	Li et al. (42) and Yang et al. (43)
Suboccipital musculature atrophy	Injection of Botulinum Toxin Type A (BTX-A) into the suboccipital musculature	8w SD male rats	As above	As above	Yang et al. (43)
Suboccipital musculature severe	After cutting the attachment points of the RCP muscles on the bone, and electrocoagulation was used to stop bleeding	8w/12w SD male rats	As a control group for suboccipital musculature hyperplasia and atrophy, it helps eliminate the physiological function of the suboccipital musculature, which more accurately explains the importance of the role of MDBC.	As above	Li et al. (42), Yang et al. (43)
Hyperplastic changes of the MDBC	Bleomycin was injected into the posterior atlanto-occipital interspace	8w SD male rats	This model provided support for the recent hypothesis proposed by Labuda et al. concerning the pathophysiology observed in symptomatic adult Chiari malformation Type I patients (98), and the view that there is a relationship between pathological changes of the MDBC and unexplained chronic headaches.	(1) It has not been experimentally demonstrated how the transmission of headache sensation is further activated by abnormal CSF circulation. (2) Given the complexity of the suboccipital region, there is a lack of hyperplastic studies of the MDBC regarding the posterior atlanto-axial interspace.	Song et al. (97)
Col22a1 knockdown of the MDBC	Local injection of COL22A1-Morpholino into the muscles of the posterior atlanto-occipital interspace	NF56 tadpole	The study of this model was the first to explore the potential influencing factors of MDBC development from a molecular biology perspective, and it made conjectures about the key molecules and possible molecular mechanisms involved in MDB development.	The absence of CSF-related experiments. No direct experimental evidence was provided for the functional association between MDBC and CSF circulation.	Yang Heng et al. (100)
ITGA7 knockdown of the MDBC	Localized ITGA7 knockdown in the dorsal atlanto-occipital interspace	Newborn SD rats (P0, < 12 h old, 6–8 g)	This study highlighted the role of ITGA7 as a key regulator in MDBC development. This study provided compelling evidence that MDB differentiation and maturation depend on the mechanical force generated by the suboccipital muscles.	As above. Besides, (1) the translational relevance of rodent findings to human physiology requires further validation. (2) Other ECM components (such as Col1a1, Lama1, Vimentin) have not been excluded from potentially compensatory or synergistic effects.	Zhang et al. (76)
Mkx overexpression and Mkx suppression of the MDBC	Utilized lentiviral plasmids to either knockdown or overexpress the Mkx gene	Newborn SD rats	Mkx exerted bidirectional regulation on MDBC development by modulating the TGF-β signaling pathway.	As above.	Li et al. (85)

Li et al. (42) and Yang et al. (43) demonstrated that BTX-A and ACE-031, respectively, were injected to suboccipital musculature of the SD rats to establish animal models of suboccipital musculature atrophy and hyperplasia. In addition, animal model with surgical severance of suboccipital musculature was set as the negative control group to eliminate the physiological function of the suboccipital musculature. The results of the studies indicated pathological changes of the suboccipital musculature could lead to changes of MDB transmission tension, resulting in changes in CSF pressure, secretion, and reabsorption rate (42, 43). These two kinds of models provide strong evidence for the involvement of MDBC in CSF circulation.

Song et al. (97) used adult SD rats as experimental subjects, bleomycin solution was locally injected to establish the hyperplastic model of MDBC. The morphological results of this model indicated that the fibers of the MDBC and other tissues in rat PAOiS undergo fibrotic changes, while cross-sectional area of the RCPmi also showed an increase (97). This model provides support for the hypothesis proposed by Labuda et al. (98) regarding the pathophysiology of symptomatic adult Chiari malformation type I patients. There is a relationship between changes in compliance of the anatomical structures of the craniocervical region and the compensatory proliferation of MDBC caused by these changes. Based on this mouse model, the viewpoint that there is a relationship between the pathological changes of MDBC and unexplained chronic headaches was further assayed (99). This study may provide anatomical and physiological explanations for the pathogenesis of some unexplained chronic headaches in clinical practice.

In addition, by using localized injection of Vivo Morpholino to establish a COL22A1 knockout animal model in *Xenopus laevis*, it has been demonstrated that COL22A1 plays a regulatory role in the development of MDBC in Xenopus laevis within a specific time frame (100). COL22A1 may influence genes related to muscle and tendon development or matrix integration in MDBC.

The research group led by Hong-Jin Sui has recently also applied lentiviral transduction technology to establish animal models of pathological changes of the MDBC, in order to investigate the regulatory mechanisms of certain genes during the developmental process of MDBC, such as Mkx (85), Integrin α7 (76). These studies provided compelling evidence that MDB differentiation and maturation depend on the mechanical force generated by the suboccipital muscles.

## 5 Clinical research of the MDBC

### 5.1 The association between MDBC and headaches

The relationship between MDBC and headaches has attracted considerable attention in the academic field. Numerous studies have shown that pathological changes of the MDBC may be closely associated with the occurrence and development of unexplained chronic headaches, shown in Figure 1 (6, 32, 59, 99, 101). Based on previous anatomical results, Alix and Bates (102) proposed that the fibrous connection between the suboccipital musculature and the pain-sensitive SDM in the upper cervical spine and occipital areas may provide anatomic and physiologic explanations for the cause of the cervicogenic headache. They suggested that chronic cervicogenic headaches might result from referred pain caused by irritation of cervical structures innervated by spinal nerves C1–C3 (102, 103). Additionally, it may result from the upper dura-muscular connection transmitting adverse forces from the cervical spine joint complex to the pain-sensitive SDM (1, 102). In order to validate this deduction, Hack et al. (6) surgically severed the connection between the RCPmi and the SDM in a patient with chronic headaches. The two-year follow-up showed that headache symptoms were significantly relieved, providing clear evidence for the role of MDB in headache (6). The primary reason for this is that, the adverse tension exerted on the suboccipital musculature during cervical instability and displacement is transmitted to the dura via MDB. Symptoms arise when this tension is transferred, and they subside once the tension is alleviated.

Subsequently, numerous studies based on MRI have investigated the morphological changes of the suboccipital musculature in different types of headaches, particularly the RCPmi. Fernández-de-las-Peñas et al. (104) and Xiaoman Min et al. (105) found significant atrophy in the RCP muscles of patients with chronic tension-type headaches, though whether it was a primary or secondary condition was unclear. Yuan et al. (101) compared the long-axis cross-sectional area of the RCPmi between patients with chronic headaches and healthy volunteers, discovering that the RCPmi in patients with chronic headache was significantly larger than that in healthy individuals. It was supposed that RCPmi hypertrophy may be one pathogenesis of the chronic headaches. Hvedstrup et al. (106) showed no difference in RCPmi volume between migraine patients and normal controls, indicating that migraine patients may be no structural pathological changes in the RCPmi. These studies show that different investigations of the suboccipital musculature, using varying morphometric measurements and methods for different headache types, have led to differing results. The reasons for these contradictory results may come from the types of headaches, sample size, methods and software used for taking measurements, and the diagnostic imaging equipment used. Of course, patient characteristics, such as age, gender, frequency, and severity of headaches, have also been considered as factors related to these results. Therefore, Hermosa et al. (32) conducted a systematic review on the morphometric alterations of the suboccipital musculature associated with primary headaches. Based on the studies included, it was concluded that there are morphometric alterations of the suboccipital musculature in patients with primary headache compared with individuals without primary headaches. However, the magnitude or direction of these alterations remains unclear. Furthermore, the impact of these morphometric changes on the pathogenesis of primary headaches is not yet fully understood.

To further investigate the relationship between pathological changes of the MDBC and the occurrence of chronic headache, animal models of the MDBC lesions were successively established. (For detailed research, refer to “4.3 Animal models for validating MDBC function.”) These models were used to explore how pathological changes of the MDBC contribute to the development of chronic headaches. Intervention on the MDBC, such as injecting BTX-A into the RCP muscles, severing the connections between the RCP muscles and the SDM, or injecting bleomycin into MDB fibers, resulted in headache-related behavioral abnormalities in rats (99). Additionally, there was a high expression of pain-related neurotransmitters in brain nuclei related to pain transmission and regulation. These findings directly demonstrate that pathological changes of the MDBC were a contributing factor to headaches. A possible underlying mechanism of chronic headaches induced by hyperplastic changes of the MDBC are as follows (99): When the MDBC and PAOM have hyperplastic changes, the compliance of the upper cervical tissues is reduced; the SDM cannot expand, and the CSF flow pattern is changed, resulting in a series of abnormalities in CSF circulation, such as abnormal CSF flow rate, velocity, and pressure. The abnormal CSF circulation may activate neural pathways in the brain, those associated with facial and head pain pathway, as well as emotion-related pathways.

### 5.2 The association between MDBC and other diseases

#### 5.2.1 MDBC and Chiari malformation type I

Chiari malformation Type I (CMI) is a serious neurological disorder characterized by herniation of the cerebellar tonsils through the foramen magnum at the cranio-cervical junction. Compromised CSF dynamics at the cranio-cervical junction is a key feature of its pathophysiology, shown in Figure 1 (107, 108). The role of the MDBC in CMI represents a recent advancement in understanding this condition.

Labuda et al. (98) have proposed a new hypothesis that clinical symptoms in symptomatic adult CMI patients may result from the combined effect of CSF restriction from herniated tonsils and reduced cervical compliance. Specifically, abnormal atlanto-occipital and/or atlanto-axial joint morphology contributes to chronic cervical instability in most CMI patients. In turn, this can lead to compensatory overactivation of the suboccipital musculature and mechanical overload and even failure of the MDBC. Over time, this will alter the mechanical properties of the SDM connected to the MDBC, making it stiffer and reducing the overall cervical compliance. The combination of restricted CSF and decreased compliance leads to elevated intracranial pressure, which exerts pressure on the cerebellum, brainstem, and upper spinal cord. Over time, this prolonged abnormality ultimately leads to microstructural damage and leads to symptoms such assecondary Valsalva-induced headache, neck pain, balance issues, abnormal sensations, and brainstem/cranial nerve issues.

In addition, denervation of the RCPmi is a neglected factor contributing to the clinical symptoms of symptomatic CMI (109). Denervation damage and reduced contractility of RCPmi is induced by long-term pathological factors such as the compression of the C1 nerve root by cerebellar tonsil herniation and the release of inflammatory mediators from the cranio-cervical junction area (109). While the MDB fibers and SDM in the cranio-cervical junction, under compression friction by the cerebellar tonsils and inflammatory factor infiltration, gradually thicken and loses compliance (109). Because of this denervation and reduced compliance, RCPmi and MDB fibers contract weakly, leading to insufficient CSF flow at the cranio-cervical junction, further increasing the pressure differential at the cranio-cervical junction, pushing the cerebellar tonsils further downward, and exacerbating the compression on the C1 nerve roots (109). The sensory fibers of the C1 nerve roots then radiate this stimulation to the cranio-cervical region, forming the typical Valsalva headache in CMI. Beyond these headaches, the sustained disturbance of CSF dynamics may lead to more severe complications, most notably syringomyelia. If CSF flow at the cranio-cervical junction is severely obstructed, this is considered a key initiating factor in the development and progression of syringomyelia. It is now widely accepted that the formation of syringomyelia is multifactorial, involving not only CSF obstruction at the cranio-cervical junction (110) but also the interplay of abnormal intracranial-spinal pressure gradients (111), altered spinal cord subarachnoid space compliance (112), and fluid dynamics within the spinal cord central canal (113). The above speculated mechanisms indicate that MDBC may play an important role in the pathophysiological process of CMI. Abnormalities in the structure and function of the MDBC could exacerbate the clinical symptoms of CMI by affecting the flow of CSF and disrupting the mechanical balance at the cranio-cervical junction (114).

In addition, as a neglected structure, MDB damage may be one of the main causes of postoperative CSF leakage and headache (114, 115). The posterior wall of the SDM at the craniovertebral junction is a multi-layered structure composed of three different originated fibers: the spinal dura mater, the periosteum located at the brim of the foramen magnum, and MDB (47). Among these, MDB fibers merged into the SDM and became part of the SDM in the atlanto-occipital and atlanto-axial interspace (18). Thus, the direct connection between MDB, PAOM, and SDM will be beneficial to the choice of surgical approach at the cranio-cervical region and the protection of the SDM, and structurally contribute to transmitting forces at the cranio-cervical junction of the CMI. However, it is important to note that traditional posterior fossa decompression procedures often lead to MDB damage and impair its ability to relieve tensile stress concentration on the dura, which can lead to pseudomeningocele or CSF leaks after surgery (116). The treatment focus of symptomatic CMI lies in restoring the normal CSF dynamics at the cranio-cervical junction. A retrospective study of CMI patients introduced a modified surgical approach. This technique, centered on protecting and reinforcing the MDB, is based on the conventional surgical procedures that these patients underwent—specifically, posterior fossa decompression with duraplasty and tonsillar coagulation (116). The key technical steps include preserving the insertions of the RCPmi and the C1 posterior arch, followed by a midline dural incision whose lower edge is sutured to the RCPmi. By protecting the PAOM and MDB, and strengthening the MDB at the incision's edge, CSF leak caused by high tension in the suture edges could be effectively avoided. Moreover, the new approach minimized the extent of the procedure and reduced the volume of the postoperative epidural space. This method provided sufficient intradural decompression, which supported normal CSF flow. This may offer a potential mechanism for improving outcomes and reducing the incidence of complications, such as decreased CSF leakage and chronic headache occurrence.

#### 5.2.2 MDBC and cervicogenic dizziness

The suboccipital musculature acts as a motion stabilizer and posture controller for the head and upper cervical joints (117). When there are changes in the structure and function of the muscles under the pillow, it may lead to dizziness, shown in Figure 1. In particular, the activation of myofascial trigger points and MDB stimulation induced by abnormal head postures may be neglected factors in cervicogenic dizziness (118).

Abnormal head postures, particularly forward head posture, can lead to altered alignment and excessive loading of the upper cervical spine (118, 119). When the head protrudes forward, the lower cervical spine flexes while the upper cervical spine extends to accommodate horizontal gaze in forward head posture (120). Correspondingly, a forward protrusion of the head has been postulated to increase anterior loading of the articular facets, leading to extension of both the occipito-atlantal and atlanto-axial joints with shortening of the RCP muscles (119, 121). The cervical extensors and occipital flexors are lengthened, while the cervical flexors and occipital extensors are shortened. Among them, the greatest shortening occurs in the suboccipital musculature (120). Therefore, the suboccipital musculature is of great priority. In addition to that, it was found that the electromyographic activity collected from the RCP muscles was increased as the head voluntarily moved from a self-selected neutral position to an extended position (119). These results indicated RCP muscles may contribute to segmental stabilization of the occipito-atlantal and atlanto-axial joints by helping to maintain joint congruency during movement of the head. However, changes in muscle length caused by long-term postural changes affect the binding between actin and myosin filaments, resulting in changes in muscle strength and endurance (122). In addition, due to instability of ligaments and facet joints, unnecessary stimulation may persist, creating a vicious cycle (119). This will lead to changes in the structure and function of surrounding muscles, especially the suboccipital musculature. Changes in the structure and function of the suboccipital musculature make it impossible to maintain constant tension of the MDB, leading to increased tension in the SDM, alterations in CSF flow, and changes in sensory and motor functions (42, 49, 123).

In addition to structural and functional abnormalities in the upper cervical spine, abnormal head posture can also impair proprioceptive function in the upper cervical region (124). Incorrect proprioceptive input may, in turn, increase muscle activation (125). Trigger points induced by excessive contraction of the suboccipital musculature can lead to proprioceptive dysfunction and nociceptor activation (123). These changes will transmit abnormal proprioceptive input to the central nervous system, and then sensory inputs are inconsistent between the vestibular and visual systems during multi-level information integration (55). It will end up with a variety of clinical symptoms of tension, including dizziness, pain, top-heavy and headache. However, not all patients with abnormal head and neck posture will experience dizziness. If patients with abnormal head and neck posture exhibit no issues with the vestibular system, cardiovascular system, or nervous system, it is necessary to investigate structural and functional problems related to MDBC.

#### 5.2.3 MDBC and Ehlers-Danlos syndrome

Ehlers-Danlos syndrome (EDS) is a group of heterogeneous hereditary connective tissue disorders characterized by joint hypermobility, connective tissue fragility, and skin extensibility (126). While most EDS patients have near-normal life spans, they often suffer from debilitating features such as pain, fatigue, and headaches. Castori et al. proposed that structural abnormalities of the MDB may contribute to the cervicogenic headaches associated with EDS (127). Subsequently, pathological structural changes of the MDB fibers, shown in Figure 1, including loosely packed collagen fibrils, abnormalities in fibril orientation, and variation in fibril size and shape, were observed in transmission electron microscopy studies of MDB fibers from EDS patients and horse models simulating EDS (21, 128). These ultrastructural changes indicated the rupture of collagen fibrils under mechanical overload of the tendon (129). As a result, the MDB fibers and other ligaments at the cranio-cervical junction may become loose, which in turn triggers spinal cord motor dysfunction (128). These changes may be an important pathogenesis of chronic neck pain and headaches in patients in EDS patients, even if no obvious instability at the cranio-cervical junction is found on imaging.

## 6 Discussion and future directions

This review presents the current knowledge of the morphology, physiology, developmental biology and pathology of MDBC.

**Figure 2:**
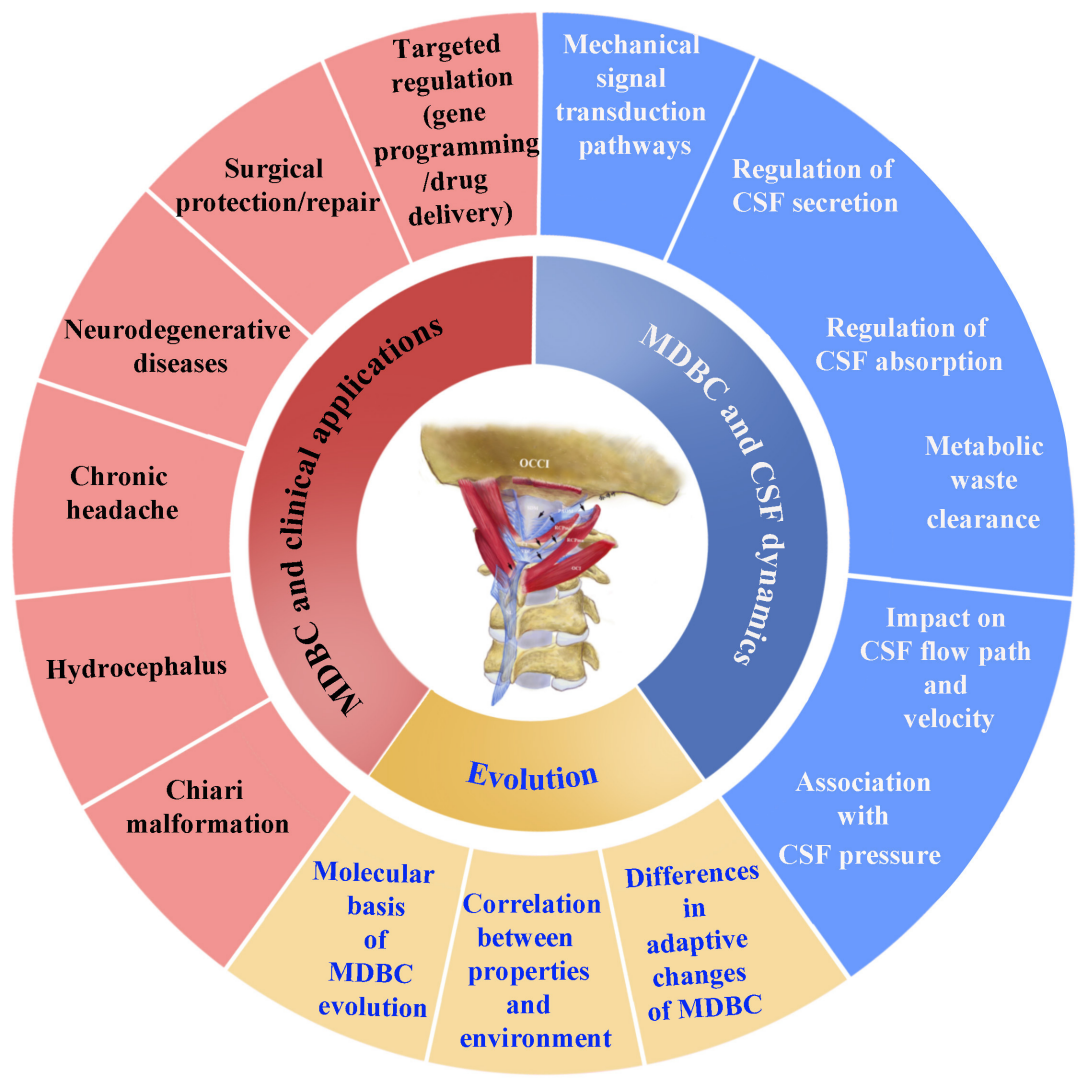
Future research directions for the myodural bridge complex (MDBC). MDBC, myodural bridge complex; CSF dynamics, cerebrospinal fluid dynamics.

Future research should clarify the adaptive variations of MDBC across vertebrates, thereby elucidating its pivotal role in driving biodiversity formation and facilitating environmental adaptation. Integrating finite element analysis with *in vitro* tissue assays will enable the validation of correlations between MDBC mechanical properties and interspecific ecological demands. Additionally, screening for MDBC development-related genes via transcriptome and single-cell sequencing—coupled with cross-species genomic comparisons—will clarify the molecular underpinnings governing the adaptive evolution of MDBC (see Figure 2).

Existing studies have demonstrated that the MDBC is capable of mechanical force transduction, which can be attributed to its primary composition of type I collagen fibers. However, most previous research has only reported morphological findings, with scarce attention paid to its mechanical properties. Specifically, the longitudinal or transverse tensile forces generated by different head movements remain to be clearly defined. This is fundamental to understanding the functional mechanism of the MDBC

The MDBC exhibits mechanical conduction properties. However, studies on its mechanisms underlying the regulation of cerebrospinal fluid circulation remain limited. Although current studies have observed changes in CSF secretion, absorption rate, and pressure following pathological changes in the MDBC, the specific mechanisms by which the MDBC influences CSF secretion, absorption, clearance, and pressure regulation remain unclear. Therefore, future research should employ diverse experimental approaches to elucidate these mechanisms.

With regard to CSF clearance, the glymphatic system is a specialized lymphoid network that facilitates the dynamic circulation of the CSF and the brain interstitial fluid and serves to eliminate metabolic waste and toxic proteins from the central nervous system (130). Studies have demonstrated that CSF enters the brain parenchyma via the paravascular space surrounding the perforating arteries, and that the brain interstitial fluid is cleared through the paravenous drainage pathways, which connect to the deep cervical lymph nodes with the nasopharynx and craniocervical region acting as critical outflow conduits (131, 132). Several factors have been identified to modulate glymphatic function, including arterial pulsations (133–138), arterial dilations during functional hyperemia (139, 140), aquaporin 4 (131), sleep (141), circadian rhythm (142), and anesthesia (143). The MDB, a key anatomical structure connecting the deep cervical muscles to the SDM, may exert an indirect regulatory effect on glymphatic system activity, which could be closely associated with the occurrence and progression of neurodegenerative diseases such as Alzheimer's disease. Normal CSF dynamics mediated by the MDB may support efficient glymphatic solute transport by maintaining optimal CSF flow velocity and perivascular space permeability. Conversely, MDB dysfunction (e.g., fibrous structural disorder, abnormal tension caused by suboccipital muscle hypertrophy/atrophy) may disrupt CSF hydrodynamics in the craniocervical region, thereby impairing the glymphatic clearance of neurotoxic proteins such as β-amyloid and tau. In addition, the MDB may modulate its own tension in response to postural changes, directly altering the compliance of the dural sac, tissue stress, and local spatial architecture in the atlanto-occipital region. This, in turn, may regulate CSF flow rate, pressure, and affect CSF production, reabsorption, and the efferent pathway of drainage into the deep cervical lymphatic system. Notably, direct experimental evidence supporting the regulatory role of the MDB in the glymphatic pathway remains limited, and further comprehensive in-depth studies are required to verify the hypothesized association between MDB function and glymphatic activity.

Its regulation of CSF pressure also requires in-depth investigation. To assess the influence of MDBC on CSF pressure, fiber optic pressure sensors can be utilized to measure spatiotemporal pressure variations across multiple sites, including intracranial pressure, subarachnoid pressure gradient, craniocervical junction, atlanto-occipital cistern and the perisinus region of dural venous sinuses. Concurrently, attention should be directed to the changes in mechanosensitive ion channels, aquaporin 1 and Na^+^/K^+^-ATPase transporters in choroid plexus epithelial cells, which may be induced by MDBC-mediated mechanical tension on the dural-vascular network. These molecular mechanisms may mediate the MDBC regulation of the homeostasis of CSF circulation.

The functional regulation of the MDBC involves two dimensions: postural changes and structural alterations. First, further research is needed to investigate the relationship between morphological parameters of the MDBC (length and tension) under different body positions and CSF velocity/direction in the cisterna magna, fourth ventricular outlet, and spinal canal. This may reveal the underlying mechanisms of CSF circulation disorders. Second, understanding how structural changes at the craniocervical junction regulate CSF spatial distribution and dynamic flow has significant clinical value for the diagnosis of related disorders. By influencing the compliance of the craniocervical junction and CSF flow, the MDBC may be implicated in CSF hydrodynamic disturbances associated with congenital abnormalities such as CMI and hydrocephalus.

In addition to regulating CSF circulation, the MDBC may also be involved in pain transmission. Animal studies have confirmed an association between the MDBC and chronic headache. However, the underlying mechanism—particularly whether the MDBC transmits pain signals via the trigeminocervical complex—remains to be verified. From a translational perspective, conservative approaches targeting the craniocervical region and the MDBC have also been explored in preliminary case-based reports. Although current evidence remains limited and cannot be generalized, recognizing these exploratory efforts offers a more balanced translational outlook. Future research frameworks may consider integrating such conservative strategies, including pharmacological interventions or physical rehabilitation, as adjuncts to surgical management. It is important to emphasize, however, that conservative treatment does not replace surgical intervention in progressive or severe cases, where mechanical decompression remains essential.

## 7 Conclusion

Comprehensive research on the morphology, comparative anatomy, development, molecular regulatory mechanisms, physiological functions, and clinical applications of MDBC reveals the importance of this structure. MDBC is a key structure universally present in vertebrates. Through specialized morphological and functional changes during the evolutionary process, MDBC achieves adaptation to environmental conditions. Furthermore, MDBC also functions as an important regulator of CSF dynamics. Despite these advances, there are still key gaps: the molecular mechanisms of MDBC development in vertebrates, the pathways through which MDBC mediates tension transduction in the CSF dynamics, and the links between MDBC pathological changes and related diseases. Future research into these gaps will not only deepen our understanding of MDBC evolution and physiology, but also accelerate its translation into clinical practice-ultimately bringing new hope to patients with diseases related to CSF dynamics or the cranio-cervical junction. In summary, multidisciplinary research on MDBC will enrich our understanding of vertebrate developmental biology and emphasize its importance as a source of CSF dynamics.
